# Urinary Sodium Excretion and the Risk of Prevalent Anemia: Nationwide Population-Based Cross-Sectional Study

**DOI:** 10.2196/88408

**Published:** 2026-04-21

**Authors:** Sang Heon Suh, Dohyeon Lee, Seong Kwon Ma, Sunyong Yoo, Soo Wan Kim

**Affiliations:** 1 Department of Internal Medicine Chonnam National University Medical School Gwangju Republic of Korea; 2 Department of Internal Medicine Chonnam National University Hospital Gwangju Republic of Korea; 3 Department of Intelligent Electronics and Computer Engineering Chonnam National University Gwangju Republic of Korea; 4 Research and Development Center MATILO AI Incorporated Gwangju Republic of Korea

**Keywords:** anemia, sodium, natriuresis, kidney, urine

## Abstract

**Background:**

While excessive dietary sodium intake is an established risk factor for cardiovascular and renal complications, its potential association with anemia remains largely unexplored.

**Objective:**

We hypothesized that, based on the observed benefits of sodium-glucose cotransporter 2 inhibitors in correcting anemia through alterations in renal tubular metabolism and oxygen homeostasis, elevated urinary sodium excretion, as a surrogate of dietary sodium intake, may increase the risk of prevalent anemia.

**Methods:**

This nationwide cross-sectional study analyzed 54,802 adults from the Korea National Health and Nutrition Examination Survey (2014-2023). Participants were stratified by spot urine sodium-to-creatinine ratio (Na^+^/Cr) quartiles (first quartile [Q1], second quartile [Q2], third quartile [Q3], and fourth quartile [Q4]). Anemia was defined as hemoglobin <13 g/dL for men and <12 g/dL for women.

**Results:**

Anemia prevalence increased progressively across spot urine Na^+^/Cr quartiles (Q1: 925/13,700, 6.8%, Q2: 1126/13,701, 8.2%, Q3: 1393/13,701, 10.2%, and Q4: 1893/13,700, 13.8%). Multivariable logistic regression demonstrated that participants in the highest quartile had 43% higher odds of anemia compared with the lowest quartile (adjusted odds ratio 1.429, 95% CI 1.269-1.610; *P*<.001). Each 1-unit log increase in spot urine Na^+^/Cr conferred a 67% increase in odds of anemia (adjusted odds ratio 1.674, 95% CI 1.452-1.930; *P*<.001). Sensitivity analyses using tertiles, quintiles, estimated 24-hour sodium excretion, and restriction to preserved kidney function consistently confirmed these associations.

**Conclusions:**

Higher urinary sodium excretion exhibits a robust, graded association with increased anemia prevalence in the general population. These findings suggest that dietary sodium restriction may provide additional benefits beyond cardiovascular protection.

## Introduction

Anemia affects more than 1.6 billion people globally and significantly impacts health care use and patient outcomes [1,2]. Beyond well-established causes, including nutritional deficiencies, chronic diseases, and genetic disorders, emerging evidence suggests that dietary factors may play broader roles than previously recognized [3].

Dietary sodium intake has received considerable attention primarily for its cardiovascular and renal implications. Current evidence firmly establishes excessive sodium consumption as a risk factor for hypertension, stroke, and chronic kidney disease [4,5]. However, potential relationships between sodium homeostasis and hematologic parameters have received limited investigation.

Recent observations from sodium-glucose cotransporter-2 (SGLT2) inhibitor trials provide intriguing insights. These medications, which increase urinary sodium excretion by inhibiting proximal tubular sodium reabsorption, consistently demonstrate increases in hemoglobin levels [6,7]. While traditionally attributed to hemoconcentration from volume contraction, this association raises questions about more direct relationships between sodium handling and erythropoiesis. Animal studies further suggest that sodium balance may influence iron metabolism and erythropoietin responsiveness [8,9].

Despite these observations, population-based evidence examining associations between sodium excretion and anemia remains scarce. Understanding such relationships could have important public health implications, particularly given ongoing efforts to reduce population sodium intake for cardiovascular protection. Therefore, we investigated the association between urinary sodium excretion and anemia prevalence using data from the Korea National Health and Nutrition Examination Survey (KNHANES), a nationally representative cross-sectional survey. We hypothesized that higher urinary sodium excretion would be associated with increased anemia prevalence, independent of traditional risk factors.

## Methods

### Study Design

**Figure 1:**
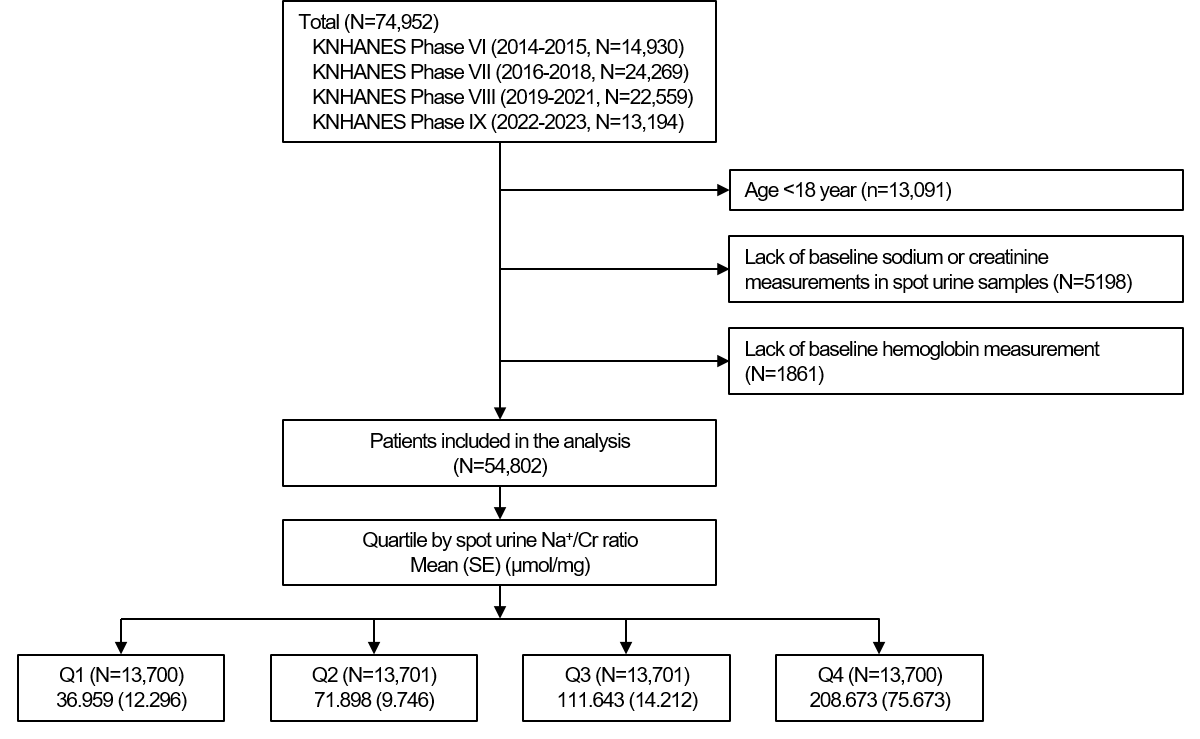
Study design. KNHANES: Korea National Health and Nutrition Examination Survey; Na+/Cr: sodium-to-creatinine ratio; Q1: first quartile; Q2: second quartile; Q3: third quartile; Q4: fourth quartile.

We analyzed data from the KNHANES, an ongoing nationwide cross-sectional survey conducted by the Korea Centers for Disease Control and Prevention to monitor the health and nutritional status of the Korean population [10]. The KNHANES uses a stratified, multistage, probability sampling design based on household registries to select a representative sample of the noninstitutionalized Korean civilian population [11]. All analyses incorporated the survey sampling weights, strata, and primary sampling units. Sampling weights reflected the inverse probability of selection with adjustments for nonresponse and poststratification to produce nationally representative estimates of the noninstitutionalized Korean population. We applied the integrated sampling weights to account for participants who completed all 3 survey components, including the nutrition survey. This study analyzed data obtained from the KNHANES 2014 to 2023 (Figure 1), as these were the most recent 10-year data available and included the measurement of spot urine sodium and creatinine. After excluding those aged less than 18 years (13,091/74,952, 17.5%) and those lacking the baseline measurement of spot urine sodium or creatinine (5198/74,952, 7%) or hemoglobin (1861/74,952, 2.5%), a total of 54,802 (73.1%) were finally included for further analyses.

### Ethical Considerations

This study protocol was approved by the Institutional Review Board of Chonnam National University Hospital (CNUH-EXP-2025-369) and was conducted in accordance with the Declaration of Helsinki and its later amendments or comparable ethical standards. All participants provided written informed consent. Identifying details of the participants were omitted. No additional compensation was provided for participation in this study.

### Exposure Assessment: Urinary Sodium Excretion

Spot urine specimens were collected during health examination visits and immediately refrigerated until measurement of urinary sodium and creatinine concentrations. We calculated the urinary sodium-to-creatinine ratio (Na^+^/Cr) as the primary exposure variable. Study participants were divided into quartiles by spot urine Na^+^/Cr (first quartile [Q1], second quartile [Q2], third quartile [Q3], and fourth quartile [Q4]; Figure 1).

### Outcome Definition: Anemia

Anemia was defined according to World Health Organization criteria: hemoglobin concentration <13.0 g/dL in men and <12.0 g/dL in nonpregnant women [12].

### Covariates

We selected potential confounders based on established associations with both sodium excretion and anemia. Variables were categorized as follows.

Demographic factors included age, sex, educational status, and household income levels.

Lifestyle factors included smoking status, drinking history, daily iron and protein intake by 24-hour recall survey, and BMI.

Clinical factors included diabetes mellitus (DM), hypertension, dyslipidemia, fasting glucose, systolic blood pressure, estimated glomerular filtration rate (eGFR), and dipstick urine protein positivity.

Comorbid conditions were operationally defined, as previously described [13,14]. DM was defined as serum fasting glucose level ≥126 mg/dL, use of antidiabetic medication, or a physician diagnosis of DM; hypertension was defined as systolic blood pressure ≥140 mm Hg, diastolic blood pressure ≥90 mm Hg, or use of antihypertensive medication; and dyslipidemia was defined either by self-report or physician diagnosis. Laboratory tests were conducted in the central laboratory (Neodin Medical Institute, Seoul, Republic of Korea) using blood samples after at least an 8-hour overnight fasting. eGFR was calculated from serum creatinine level using the Chronic Kidney Disease Epidemiology Collaboration equation [15]. Proteinuria was defined as albuminuria (≥1+) determined by dipstick urine test [13].

### Statistical Analysis

Continuous and categorical variables were expressed as the mean (SE) and n (%), respectively. Baseline characteristics across quartiles were compared using survey-weighted ANOVA for continuous variables and chi-square test for categorical variables. To assess the shape of the association between spot urine Na^+^/Cr and hemoglobin, restricted cubic splines were fitted using multivariable linear regression [16]. Multivariable associations with anemia were estimated using survey-weighted logistic regression, accounting for strata, clusters, and sampling weights, and are reported as adjusted odds ratios (ORs) with 95% CIs [17]. ORs were used as the primary measure of association, as they allow for robust adjustment of multiple confounders. Given that the prevalence of anemia in most of our study population was <10%, the OR served as a reliable approximation of the prevalence ratio according to the rare disease assumption. Participants with any missing values were excluded from the primary analysis, while missing values were handled using multiple imputation according to variable types in a sensitivity analysis. For each variable with missing data, 5 imputed datasets were generated. Categorical variables were imputed using multinomial logistic regression, and continuous variables were imputed using the predictive mean matching method. Statistical analyses were conducted separately on each imputed dataset, and the results were pooled using the Rubin rule [18]. This approach appropriately accounts for the uncertainty introduced by missing values while maintaining the validity of statistical inference [19]. All statistical analyses were performed in SAS (version 9.4; SAS Institute; survey procedures), and figures were generated in R (version 4.4.1; R Foundation for Statistical Computing), with statistical significance defined as *P*<.05.

## Results

### Baseline Characteristics

The baseline characteristics of the participants across the quartiles by spot urine Na^+^/Cr demonstrated significant graded differences in demographic, anthropometric, and laboratory parameters (Table 1). Individuals in the highest quartile (Q4) were notably older (mean 58.3, SE 0.2 vs mean 39.1, SE 0.2 years; *P*<.001) and more likely to be female participants (4347/13,700, 31.7% vs 6315/13,700, 46.1%; *P*<.001) compared with the lowest quartile (Q1). Higher spot urine Na^+^/Cr quartiles exhibited a stepwise increase in cardiovascular risk factors, including DM (2518/13,700, 18.4% in Q4 vs 953/13,700, 7% in Q1), hypertension (6363/13,700, 46.4% vs 2937/13,700, 21.4%), and dyslipidemia (4118/13,700, 30.1% vs 1604/13,700, 11.7%), with statistical significance in all the comparisons (*P*<.001). A consistent inverse relationship was observed between spot urine Na^+^/Cr and hemoglobin concentration, declining from a mean of 14.5 (SE 0.02) g/dL in Q1 to a mean of 13.6 (SE 0.02) g/dL in Q4 (*P*<.001). Concurrently, eGFR decreased progressively across quartiles (mean 104.8, SE 0.2 in Q1 vs mean 96.6, SE 0.2 mL/min/1.73 m² in Q4; *P*<.001), while proteinuria prevalence exhibited a U-shaped distribution, being highest in Q1 (605/13,700, 4.4%) and lowest in Q3 (253/13,701, 1.8%).

**Table 1:** Baseline characteristics of participants by quartiles of spot urine sodium-to-creatinine ratio (Na+/Cr).

		Q1 (n=13,700)	Q2 (n=13,701)	Q3 (n=13,701)	Q4 (n=13,700)	*P* value	
Age (y), mean (SE)		39.124 (0.156)	45.230 (0.177)	50.598 (0.185)	58.285 (0.198)	<.001	
Male, n (%)		7385 (53.9)	6804 (49.7)	5994 (43.7)	4347 (31.7)	<.001	
**Education (years), n (%)**							<.001
	≤6	1180 (8.6)	1739 (12.7)	2583 (18.9)	4451 (32.5)		
	7-9	868 (6.3)	1236 (9)	1482 (10.8)	1870 (13.6)		
	10-12	4734 (34.6)	4440 (32.4)	4436 (32.4)	3702 (27)		
	>12	6364 (46.4)	5681 (41.5)	4468 (32.6)	2780 (20.3)		
**Household income, n (%)**							<.001
	<20%	1544 (11.3)	1730 (12.6)	2275 (16.6)	3476 (25.4)		
	20%-39%	2174 (15.9)	2408 (17.6)	2545 (18.6)	3138 (22.9)		
	40%-59%	2895 (21.1)	2874 (21)	2794 (20.4)	2450 (17.9)		
	60%-79%	3369 (24.6)	3149 (23)	3017 (22)	2287 (16.7)		
	≥80%	3673 (26.8)	3485 (25.4)	3022 (22.1)	2273 (16.6)		
**Smoking history, n (%)**							<.001
	Current smoker	3241 (23.7)	2543 (18.6)	2049 (15)	1451 (10.6)		
	Former smoker	2879 (21)	3255 (23.8)	3251 (v)	2697 (19.7)		
	Nonsmoker	7580 (55.3)	7903 (57.7)	8401 (61.3)	9552 (69.7)		
Iron intake (mg/d), mean (SE)		11,995 (0.104)	12.314 (0.204)	12.093 (0.101)	11.107 (0.096)	<.001	
Protein intake (g/kg/d), mean (SE)		1.157 (0.007)	1.139 (0.007)	1.109 (0.007)	1.036 (0.007)	<.001	
**Drinking history, n (%)**							<.001
	Nondrinking	1790 (13.1)	2102 (15.3)	2523 (18.4)	2885 (21.1)		
	Occasional moderate drinking	3168 (23.1)	3443 (25.1)	3485 (25.4)	3534 (25.8)		
	Regular moderate drinking	276 (2)	346 (2.5)	354 (2.6)	428 (3.1)		
	Occasional binge drinking	2552 (18.6)	2175 (15.9)	1785 (13)	1266 (9.2)		
	Regular binge drinking	4839 (35.3)	4319 (31.5)	3765 (27.5)	2776 (20.3)		
**Medical history, n (%)**							
	DM^a^	953 (7)	1266 (9.2)	1648 (12)	2518 (18.4)	<.001	
	Hypertension	2937 (21.4)	3693 (27)	4546 (33.2)	6363 (46.4)	<.001	
	Dyslipidemia	1604 (11.7)	2318 (16.9)	2993 (21.8)	4118 (30.1)	<.001	
BMI (kg/m^2^), mean (SE)		23.852 (0.039)	24.047 (0.038)	24.156 (0.040)	24.110 (0.036)	<.001	
SBP^b^ (mm Hg), mean (SE)		114.128 (0.140)	116.357 (0.155)	118.856 (0.170)	123.884 (0.201)	<.001	
**Laboratory findings**							
	Hemoglobin (g/dL), mean (SE)	14.535 (0.016)	14.283 (0.015)	14.014 (0.016)	13.595 (0.016)	<.001	
	Fasting glucose (mg/dL), mean (SE)	97.495 (0.220)	99.207 (0.219)	101.437 (0.241)	103.852 (0.259)	<.001	
	Total cholesterol (mg/dL), mean (SE)	191.102 (0.384)	191.251 (0.392)	191.036 (0.395)	189.003 (0.412)	<.001	
	HDL-C^c^ (mg/dL), mean (SE)	52.445 (0.138)	52.441 (0.142)	52.599 (0.137)	53.230 (0.145)	<.001	
	eGFR^d^ (mL/min/1.73 m^2^), mean (SE)	104.775 (0.170)	102.736 (0.181)	100.217 (0.186)	96.556 (0.202)	<.001	
	Dipstick urine protein ≥1+, n (%)	605 (4)	318 (2.2)	253 (1.6)	282 (1.9)	<.001	

^a^DM: diabetes mellitus.

^b^SBP: systolic blood pressure.

^c^eGFR: estimated glomerular filtration rate.

^d^HDL-C: high-density lipoprotein cholesterol.

### Association of Urinary Sodium Excretion and the Risk of Prevalent Anemia

**Figure 2:**
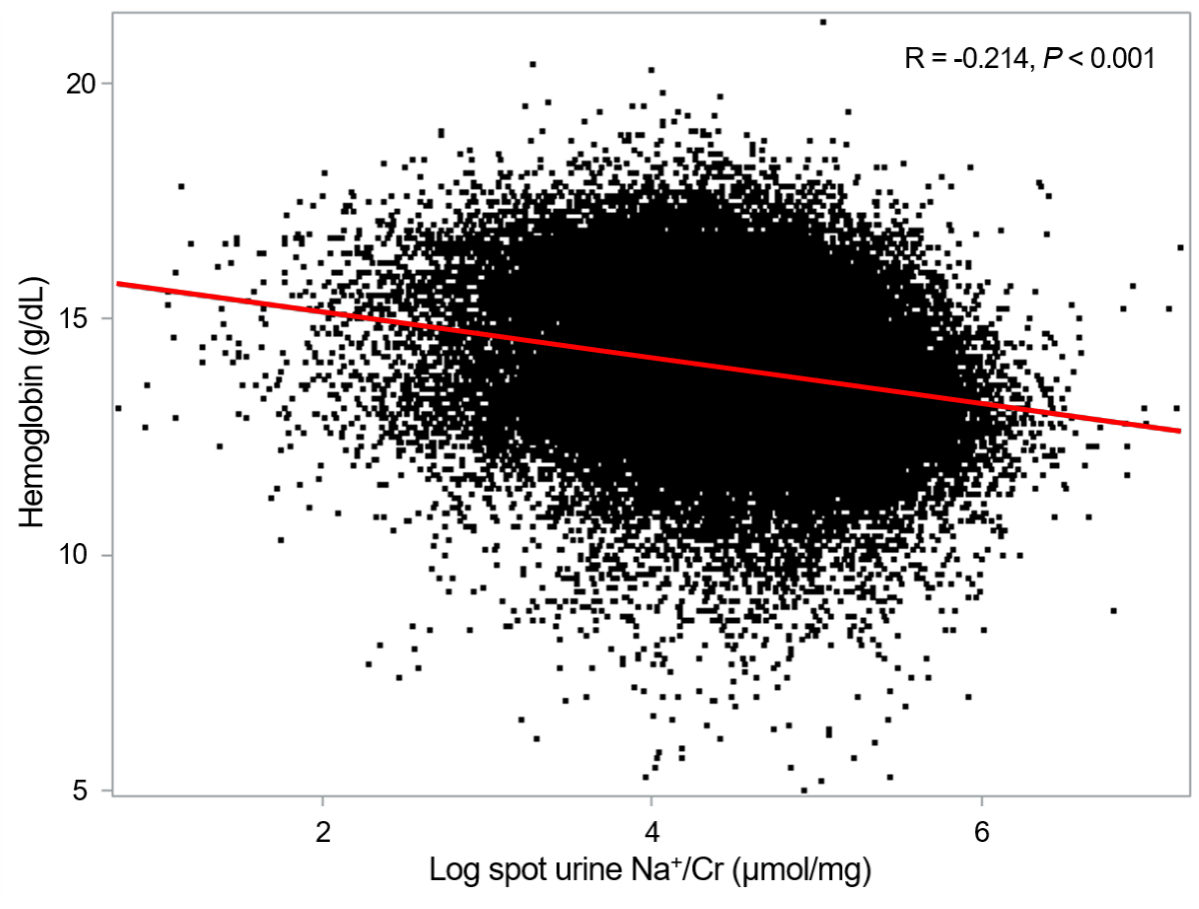
Scatter plot of spot urine sodium-to-creatinine ratio (Na+/Cr) with hemoglobin levels. The correlation of spot urine Na+/Cr with hemoglobin levels was assessed by the Pearson correlation coefficient (r). A linear inverse correlation was observed between spot urine Na+/Cr and hemoglobin levels, though the correlation intensity is low.

**Figure 3:**
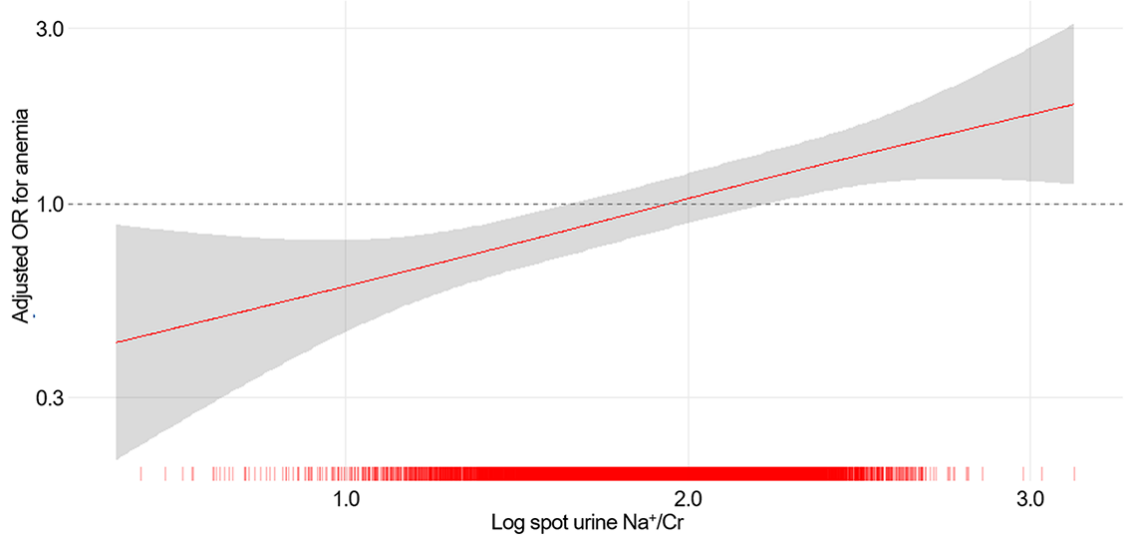
Penalized spline curve of spot urine sodium-to-creatinine ratio (Na+/Cr) and the risk of prevalent anemia. Adjusted for age, sex, household income levels, education years, smoking history, medical history (diabetes mellitus, hypertension, and dyslipidemia), BMI, systolic blood pressure, fasting glucose, estimated glomerular filtration rate, and dipstick urine protein positivity. A linear association was observed between spot urine Na+/Cr and the risk of prevalent anemia. OR: odds ratio.

A clear dose-response relationship emerged between spot urine Na^+^/Cr and anemia prevalence. The scatter plot analysis revealed a significant negative correlation between spot urine Na^+^/Cr and hemoglobin concentration (*r*=−0.214; *P*<.001; Figure 2). The prevalence of anemia increased progressively across quartiles from 6.8% (925/13,700) in Q1 to 13.8% (1893/13,700) in Q4. Multivariable logistic regression analysis demonstrated graded associations that remained significant after adjustment for potential confounders (Table 2). In the fully adjusted model (model 4), which accounted for demographic variables, comorbidities, metabolic parameters, kidney function, and proteinuria, participants in Q4 exhibited 43% higher odds of prevalent anemia compared with Q1 (adjusted OR 1.429, 95% CI 1.269-1.610; *P*<.001). Intermediate quartiles showed incrementally increasing risk, with an adjusted OR of 1.207 (95% CI 1.073-1.357; *P*=.002) in Q2 and an adjusted OR of 1.306 (95% CI 1.167-1.462; *P*<.001) in Q3. When modeled continuously, each 1-unit log increase in spot urine Na^+^/Cr was associated with 67% higher odds of anemia (adjusted OR 1.674, 95% CI 1.452-1.930; *P*<.001). The relationship between spot urine Na^+^/Cr and hemoglobin concentration was further characterized using penalized spline curve analysis (Figure S1 in Multimedia Appendix 1), demonstrating a linear inverse association. Complementary spline analysis modeling the risk of anemia as the outcome confirmed these findings, revealing a monotonic increase in ORs across the spectrum of spot urine Na^+^/Cr values after full covariate adjustment (Figure 3). The curves demonstrated no apparent inflection points, suggesting that the relationship between urinary sodium excretion and anemia risk operates continuously throughout the observed range.

**Table 2:** Association between spot urine sodium-to-creatinine ratio (Na+/Cr) and anemia prevalence: multivariable logistic regression analysis.

	Spot urine Na^+^/Cr (μmol/mg)		Prevalence, n (%)		Model 1^a^					Model 2^b^					Model 3^c^					Model 4^d^			
					OR^e^ (95% CI)		*P* value		OR (95% CI)			*P* value		OR (95% CI)			*P* value		OR (95% CI)			*P* value	
Q1^f^ (n=13,700)	2.1-54.1		925 (6.8)		Reference		—^g^		Reference			—		Reference			—		Reference			—	
Q2^h^ (n=13,701)	54.1-87.2		1126 (8.2)		1.311 (1.178-1.459)		<.001		1.092 (0.978-1.218)			.12		1.147 (1.022-1.287)			0.019		1.207 (1.073-1.357)			.002	
Q3^i^ (n=13,701)	87.2-136.7		1393 (10.2)		1.631 (1.479-1.798)		<.001		1.121 (1.012-1.242)			.03		1.210 (1.085-1.350)			<.001		1.306 (1.167-1.462)			<.001	
Q4^j^ (n=13,700)	136.7-1344.1		1893 (13.8)		2.415 (2.192-2.660)		<.001		1.225 (1.100-1.365)			.002		1.300 (1.159-1.457)			<.001		1.429 (1.269-1.610)			<.001	
Per 1 log increase	—		—		3.237 (2.865-3.657)		<.001		1.346 (1.184-1.530)			<.001		1.466 (1.277-1.683)			<.001		1.674 (1.452-1.930)			<.001	

^a^Model 1: unadjusted.

^b^Model 2: model 1 adjusted for age and sex.

^c^Model 3: model 2 adjusted for household income, education years, smoking history, medical history (diabetes mellitus, hypertension, and dyslipidemia), BMI, and systolic blood pressure.

^d^Model 4: model 3 adjusted for fasting glucose, estimated glomerular filtration rate, and dipstick urine protein positivity.

^e^OR: odds ratio.

^f^Q1: first quartile.

^g^Not applicable.

^h^Q2: second quartile.

^i^Q3: third quartile.

^j^Q4: fourth quartile.

### Sensitivity Analysis

Sensitivity analyses confirmed the robustness of the primary findings. Alternative categorization schemes using tertiles and quintiles of spot urine Na^+^/Cr yielded consistent results (Table S1 in Multimedia Appendix 1). In tertile analysis, the highest tertile demonstrated an adjusted OR of 1.415 (95% CI 1.278-1.567; *P*<.001) compared with the lowest tertile in the fully adjusted model. Quintile stratification revealed a more granular dose-response gradient, with ORs progressively increasing from 1.218 (second quintile) to 1.547 (fifth quintile) relative to the lowest quintile (all *P*<.01). When using estimated 24-hour urinary sodium excretion by the Tanaka formula [20] as an alternative exposure measure, the association remained significant, with the highest quartile exhibiting an adjusted OR of 1.376 (95% CI 1.237-1.531; *P*<.001) in model 4 (Table S2 in Multimedia Appendix 1), though the effect size was modestly attenuated compared with spot urine Na^+^/Cr. To address potential confounding by fluid retention related to high sodium intake in participants with impaired kidney function, we performed a restricted analysis excluding participants with eGFR <60 mL/min/1.73 m² (1513/54,802, 2.8%), where the association between spot urine Na^+^/Cr and anemia risk persisted with minimal attenuation (Table S3 in Multimedia Appendix 1). The highest quartile retained 27% higher odds of anemia (adjusted OR 1.266, 95% CI 1.118-1.434; *P*<.001) compared with the reference quartile after full adjustment. The association remained unchanged even after additional adjustment for daily iron and protein intake (24-hour dietary recall survey) and alcohol consumption (Table S4 in Multimedia Appendix 1). Finally, multiple imputation for missing covariate data yielded nearly identical point estimates and CIs to the complete-case analysis, with Q4 demonstrating an adjusted OR of 1.429 (95% CI 1.269-1.610; *P*<.001) in the fully adjusted model (Table 3), confirming that missing data did not materially influence the observed associations.

**Table 3:** Binary logistic regression of spot urine sodium-to-creatinine ratio (Na+/Cr) levels for the risk of prevalent anemia after multiple imputation.

Spot urine Na^+^/Cr level	Model 1^a^			Model 2^b^			Model 3^c^			Model 4^d^	
	OR^e^ (95% CI)	*P* value	OR (95% CI)		*P* value	OR (95% CI)		*P* value	OR (95% CI)		*P* value
Q1^f^	Reference	—^g^	Reference		—	Reference		—	Reference		—
Q2^h^	1.321 (1.182-1.475)	<.001	1.107 (0.988-1.240)		.08	1.148 (1.023-1.283)		.02	1.207 (1.073-1.357)		.002
Q3^i^	1.617 (1.460-1.791)	<.001	1.137 (1.021-1.266)		.02	1.211 (1.085-1.351)		<.001	1.306 (1.167-1.462)		<.001
Q4^j^	2.313 (2.091-2.558)	<.001	1.215 (1.085-1.360)		<.001	1.301 (1.160-1.458)		<.001	1.429 (1.269-1.610)		<.001

^a^Model 1: unadjusted.

^b^Model 2: model 1 adjusted for age and sex.

^c^Model 3: model 2 adjusted for household income, education years, smoking history, medical history (diabetes mellitus, hypertension, and dyslipidemia), BMI, and systolic blood pressure.

^d^Model 4: model 3 adjusted for fasting glucose, total cholesterol, estimated glomerular filtration rate, and dipstick urine protein positivity.

^e^OR, odds ratio.

^f^Q1: first quartile.

^g^Not applicable.

^h^Q2: second quartile.

^i^Q3: third quartile.

^j^Q4: fourth quartile.

## Discussion

### Main Findings

In this nationwide cross-sectional study involving 54,802 Korean adults, we identified a robust and graded association between higher urinary sodium excretion and increased prevalence of anemia. This relationship persisted after comprehensive adjustment for demographic variables, comorbid conditions, eGFR, and proteinuria, with individuals in the highest quartile of spot urine Na^+^/Cr demonstrating 43% higher odds of anemia compared with the lowest quartile. To our knowledge, this represents the first population-based investigation examining the link between dietary sodium intake and anemia risk in the general population.

### Clinical Implications

Our findings extend beyond the established cardiovascular and renal consequences of excess sodium intake. The observed association between sodium excretion and anemia prevalence—with rates increasing from 6.8% (925/13,700) to 13.8% (1892/13,700) across quartiles—represents a clinically meaningful effect size comparable to traditional anemia risk factors. These data suggest that dietary sodium reduction may be an additional option for the prevention or correction of anemia.

### Biological Plausibility

High sodium intake may increase anemia prevalence by altering cellular metabolism in renal proximal tubular cells. Sodium reabsorption constitutes the predominant energy-consuming process in the kidney, accounting for more than 90% of total renal oxygen consumption [21,22]. The proximal tubule, responsible for reabsorbing approximately 60% to 70% of filtered sodium, operates under relatively high metabolic demand despite its favorable oxygen supply from the renal cortex [23]. Studies examining high salt intake in experimental models have demonstrated progressive increases in whole-kidney oxygen consumption accompanied by upregulation of glycolytic pathway genes in proximal tubular segments, suggesting heightened metabolic stress [24]. Higher metabolic demand triggers tubulointerstitial hypoxia, compromising the function of erythropoietin-producing cells.

Our findings align conceptually with findings from previous clinical trials of SGLT2 inhibitors, in which these agents consistently increased hemoglobin levels by 0.6 to 0.7 g/dL with enhanced erythropoietin production and red blood cell mass expansion [6,7]. The proposed mechanism involved reduced proximal tubular workload through blockade of sodium-glucose reabsorption, thereby decreasing cellular ATP consumption and oxygen demand required for sodium handling [25]. This metabolic reprieve alleviates tissue hypoxia and preserves erythropoietin-producing interstitial fibroblasts by suppressing the transition to myofibroblasts under sustained tissue hypoxia [9]. Importantly, experimental studies have shown that dietary sodium restriction redistributes the tubular metabolic workload, shifting sodium reabsorption from less energy-efficient distal segments toward the more efficient proximal tubule [26]. Conversely, high dietary sodium intake shifts reabsorption distally, increasing overall oxygen consumption relative to sodium transport efficiency [26]. This metabolic framework suggests that lower dietary sodium may confer hematologic benefits through mechanisms similar to SGLT2 inhibition.

### Alternative Explanation

Hemodilution related to volume expansion from high sodium intake should also be considered as an alternative explanation. Plasma volume expansion can reduce hemoglobin concentration without decreasing red blood cell mass, thereby causing apparent rather than true anemia. However, several lines of evidence argue against hemodilution as the primary mechanism for the principal findings of this study. First, the association between urinary sodium excretion and anemia persisted after adjustment for blood pressure and BMI, both of which reflect sodium-dependent volume expansion. Second, studies examining blood volume components in various patient populations demonstrate that, while hemodilution contributes to anemia in acute fluid overload states such as heart failure, the relationship between chronic dietary sodium intake and sustained plasma volume expansion in otherwise healthy individuals is more complex [27,28]. Significant acute volume changes are unlikely, given the community-dwelling nature of the KNHANES cohort. Third, the findings from the trials of SGLT2 inhibitors suggest that, although these agents result in transient natriuresis and plasma volume contraction, the increase in hemoglobin is sustained much longer, with enhanced erythropoiesis reflected by changes in iron metabolism markers [29]. Fourth, as most participants in the KNHANES dataset have normal kidney function (ie, eGFR ≥60 mL/min/1.73 m^2^), dietary salt intake should be balanced with urinary sodium excretion rather than sodium retention in that population.

### Limitations

Several limitations should be acknowledged. First, the cross-sectional design precludes causal inference, and residual confounding from unmeasured variables remains possible despite extensive adjustment. Second, we lacked data on dietary patterns, micronutrient intake, and other nutritional factors that influence anemia risk. Third, as hemoglobin measurements occurred at a single time point, we were unable to distinguish acute from chronic anemia or assess temporal relationships between sodium intake changes and hemoglobin dynamics. Fourth, spot urine specimens, while practical for population surveys, provide less accurate sodium excretion estimates than timed collections. As 24-hour urinary sodium excretion remains the gold standard of the surrogate for dietary sodium intake, spot urine sodium excretion is less accurate. Fifth, due to the intrinsic limitation of the dataset, only cross-sectional analysis was available, which was not sufficient to suggest a causal relation between excess sodium intake and the risk of prevalent anemia. Finally, our findings were derived from a Korean population, and their applicability to other ethnic groups requires validation, considering that population-specific dietary patterns significantly differ from those of a typical Western diet.

### Conclusions

In conclusion, we demonstrate a novel association between urinary sodium excretion and the risk of prevalent anemia in a nationwide Korean population. These data suggest that, in the context of the findings from trials of SGLT2 inhibitors, excessive dietary sodium intake may impair the production of erythropoietin from a subset of kidney interstitial fibroblasts. Our findings indicate that dietary sodium restriction may reduce the risk of anemia, in addition to the established cardiovascular and renal benefits. While confirmatory studies are required, our findings support expanding the rationale for sodium restriction beyond traditional cardiovascular and renal indications to include potential hematologic benefits.

## Data Availability

The datasets generated or analyzed during this study are available in the Korea National Health and Nutrition Examination Survey (KNHANES) repository [30].

## Appendix

Multimedia Appendix 1 Additional data on the different binary logistic regressions of spot urine Na+/Cr levels.

## Abbreviations

DM: diabetes mellitus
eGFR: estimated glomerular filtration rate
KNHANES: Korea National Health and Nutrition Examination Survey
Na+/Cr: sodium-to-creatinine ratio
OR: odds ratio
Q1: first quartile
Q2: second quartile
Q3: third quartile
Q4: fourth quartile
SGLT2: sodium-glucose cotransporter 2
