# Supplementary material for: Urinary Sodium Excretion and the Risk of Prevalent Anemia: Nationwide Population-Based Cross-Sectional Study
Source: JMIR Public Health Surveill. 2026 Apr 21;12:e88408. doi: 10.2196/88408 (PMC13146232; doi:10.2196/88408)
Supplement: Multimedia Appendix 1 [file publichealth_v12i1e88408_app1.docx]

**- Supplementary Material -**

**Urinary Sodium Excretion and the Risk of Prevalent Anemia: A Nationwide Population-Based Cross-Sectional Study from the KNHANES**

Sang Heon Suh, M.D., Ph.D.^1,2,*^, Dohyeon Lee^3,*^, Seong Kwon Ma, M.D., Ph.D.^1,2^, Sunyong Yoo, Ph.D.^3,4,†^, and Soo Wan Kim, M.D., Ph.D.^1,2,†^

^1^Department of Internal Medicine, Chonnam National University Medical School, Gwangju, Republic of Korea

^2^Department of Internal Medicine, Chonnam National University Hospital, Gwangju, Republic of Korea

^3^Department of Intelligent Electronics and Computer Engineering, Chonnam National University, Gwangju, Republic of Korea

^4^R&D Center, MATILO AI Inc., Gwangju, Republic of Korea

^*^S.H.S. and D.L. equally contributed to this work as the co-first author

^†^S.Y. and S.W.K. equally contributed to this work as the co-corresponding author

**Corresponding authors:**

**Soo Wan Kim, M.D., Ph.D.**

Tel: +82-62-225-6271

Fax: +82-62-220-8578

E-mail: skimw@chonnam.ac.kr

**Sunyong Yoo, Ph.D.**

Tel: +82-62-530-1761

Fax: +82-62-530-3439

E-mail: syyoo@jnu.ac.kr

**
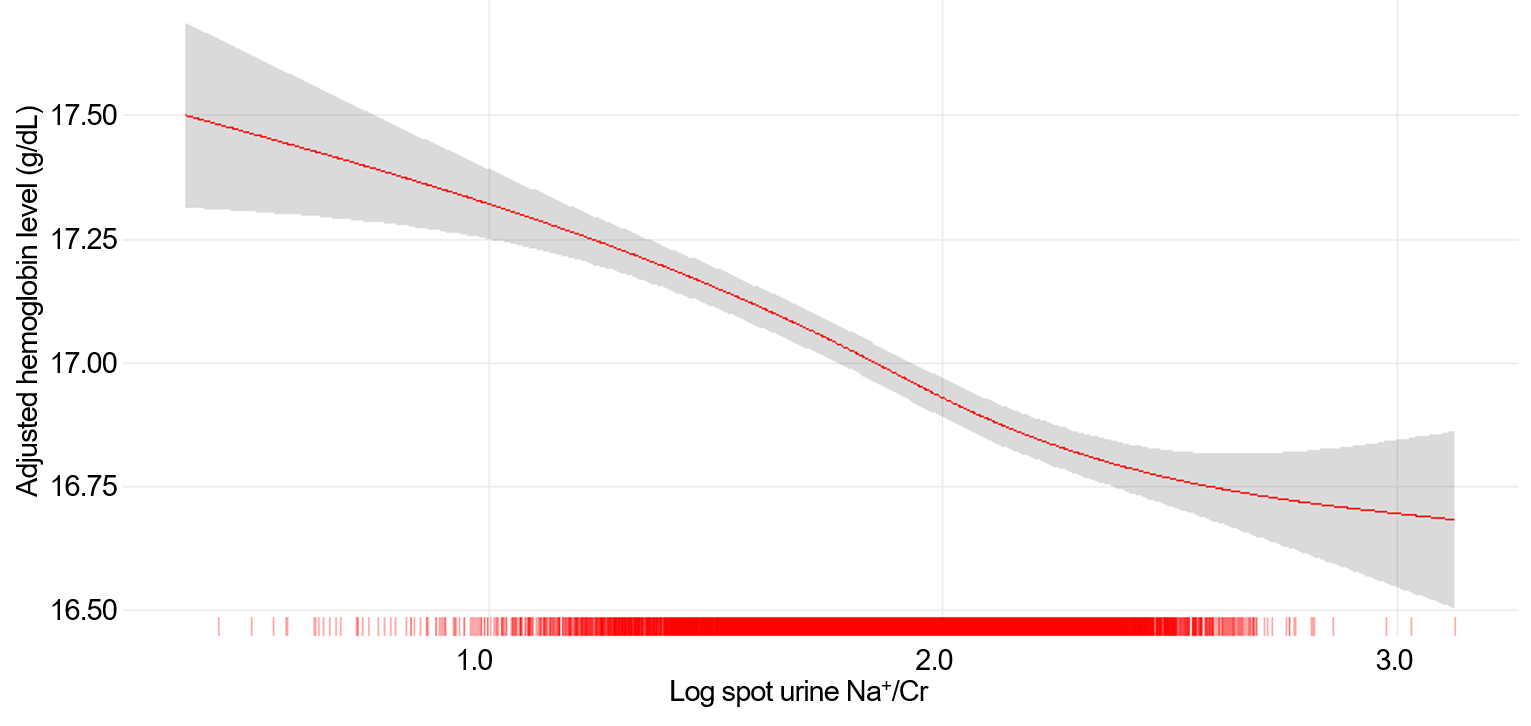
**

**Figure S1. Penalized spline curve of spot urine Na^+^/Cr on hemoglobin levels**

Adjusted for age, sex, householding income levels, education years, smoking history, medical history (DM, HTN, and DL), BMI, SBP, fasting glucose, eGFR and dipstick urine protein positivity. Note a linear inverse association between spot urine Na^+^/Cr and hemoglobin levels. Na^+^/Cr, sodium-to-creatinine ratio; OR, odds ratio.

**Table S1. Binary logistic regression of spot urine Na^+^/Cr levels divided into the tertile and quintile for the risk of prevalent anemia**

|  | Spot urine Na^+^/Cr (μmol/mg) | Prevalence, n (%) | Model 1 | | Model 2 | | Model 3 | | Model 4 | |
| --- | --- | --- | --- | --- | --- | --- | --- | --- | --- | --- |
|  |  |  | OR (95%CI) | *P* value | OR (95%CI) | *P* value | OR (95%CI) | *P* value | OR (95%CI) | *P* value |
| 1^st^ tertile | 2.1 – 64.1 | 1,265 (5.8) | Reference |  | Reference |  | Reference |  | Reference |  |
| 2^nd^ tertile | 64.1 – 114.8 | 1,645 (7.8) | 1.385  (1.271, 1.509) | < 0.001 | 1.088  (0.995, 1.188) | 0.063 | 1.154  (1.050, 1.268) | 0.029 | 1.211  (1.100, 1.333) | < 0.001 |
| 3^rd^ tertile | 114.8 – 1344.1 | 2,427 (11.9) | 2.198  (2.022, 2.390) | < 0.001 | 1.227  (1.119, 1.347) | < 0.001 | 1.306  (1.183, 1.441) | < 0.001 | 1.415  (1.278, 1.567) | < 0.001 |
| 1^st^ quintile | 2.1 – 47.6 | 713 (5.3) | Reference |  | Reference |  | Reference |  | Reference |  |
| 2^nd^ quintile | 47.6 – 73.3 | 834 (6.6) | 1.260  (1.118, 1.420) | 0.001 | 1.085  (0.960, 1.226) | 0.718 | 1.144  (1.006, 1.302) | 0.040 | 1.218  (1.068, 1.388) | 0.003 |
| 3^rd^ quintile | 73.3 – 103.5 | 1,014 (8.1) | 1.578  (1.411, 1.765) | < 0.001 | 1.171  (1.042, 1.316) | 0.437 | 1.263  (1.117, 1.429) | < 0.001 | 1.366  (1.205, 1.549) | < 0.001 |
| 4^th^ quintile | 103.5 – 151.3 | 1,203 (9.7) | 1.916  (1.711, 2.146) | < 0.001 | 1.215  (1.077, 1.371) | 0.007 | 1.311  (1.153, 1.490) | < 0.001 | 1.446  (1.268, 1.648) | < 0.001 |
| 5^th^ quintile | 151.3 – 1344.1 | 1,573 (12.9) | 2.664  (2.388, 2.972) | < 0.001 | 1.279  (1.132, 1.445) | 0.009 | 1.382  (1.213, 1.574) | < 0.001 | 1.547  (1.362, 1.770) | < 0.001 |

Model 1, unadjusted model. Model 2, model 1 + adjusted for age, sex. Model 3, model 2 + household income levels, education years, smoking history, medical history (DM, HTN, and DL), BMI, and SBP. Model 4, model 3 + adjusted for fasting glucose, eGFR and dipstick urine protein positivity. CI, confidence interval; OR, odds ratio; Na^+^/Cr, sodium-to-creatinine ratio.

**Table S2. Binary logistic regression of estimated 24h urine sodium excretion levels for the risk of prevalent anemia**

|  | Estimated 24h urine sodium (mmol/day) | Prevalence, n (%) | Model 1 | | Model 2 | | Model 3 | | Model 4 | |
| --- | --- | --- | --- | --- | --- | --- | --- | --- | --- | --- |
|  |  |  | OR (95%CI) | *P* value | OR (95%CI) | *P* value | OR (95%CI) | *P* value | OR (95%CI) | *P* value |
| Q1 | 180.4 – 685.4 | 1,199 (7.4) | Reference |  | Reference |  | Reference |  | Reference |  |
| Q2 | 685.4 – 825.1 | 1,255 (7.9) | 1.074  (0.977, 1.181) | 0.140 | 1.033  (0.937, 1.138) | 0.516 | 1.119  (1.011, 1.238) | 0.029 | 1.162  (1.049, 1.287) | 0.004 |
| Q3 | 825.1 – 977.0 | 1,309 (8.2) | 1.108  (1.006, 1.220) | 0.037 | 1.018  (0.921, 1.124) | 0.726 | 1.143  (1.028, 1.271) | 0.013 | 1.220  (1.095, 1.360) | < 0.001 |
| Q4 | 977.0 – 2611.1 | 1,467 (8.9) | 1.225  (1.116, 1.344) | < 0.001 | 1.056  (0.960, 1.162) | 0.262 | 1.279  (1.154, 1.417) | < 0.001 | 1.376  (1.237, 1.531) | < 0.001 |

Model 1, unadjusted model. Model 2, model 1 + adjusted for age, sex. Model 3, model 2 + household income levels, education years, smoking history, medical history (DM, HTN, and DL), BMI, and SBP. Model 4, model 3 + adjusted for fasting glucose, eGFR and dipstick urine protein positivity. CI, confidence interval; OR, odds ratio; Na^+^/Cr, sodium-to-creatinine ratio; Q1, 1^st^ quartile; Q2, 2^nd^ quartile; Q3, 3^rd^ quartile; Q4, 4^th^ quartile.

**Table S3. Binary logistic regression of spot urine Na^+^/Cr levels for the risk of prevalent anemia after excluding the participants with eGFR < 60 mL/min/1.73m^2^**

|  | Spot urine Na^+^/Cr (μmol/mg) | Prevalence, n (%) | Model 1 | | Model 2 | | Model 3 | | Model 4 | |
| --- | --- | --- | --- | --- | --- | --- | --- | --- | --- | --- |
|  |  |  | OR (95%CI) | *P* value | OR (95%CI) | *P* value | OR (95%CI) | *P* value | OR (95%CI) | *P* value |
| Q1 | 2.1 – 55.2 | 841 (5.3) | Reference |  | Reference |  | Reference |  | Reference |  |
| Q2 | 55.2 – 88.7 | 1031 (6.8) | 1. 310  (1.172, 1.465) | <0.001 | 1.124  (1.002, 1.260) | 0.045 | 1.171  (1.039, 1.320) | 0.001 | 1.162  (1.031, 1.311) | 0.014 |
| Q3 | 88.7 – 138.2 | 1230 (8.1) | 1.586  (1.431, 1.757) | < 0.001 | 1.149  (1.032, 1.279) | 0.011 | １。２２６  (1.094, 1.374) | < 0.001 | 1.219  (1.085, 1.368) | < 0.001 |
| Q4 | 138.2 – 1344.1 | 1610 (11.2) | 2.254  (2.034, 2.498) | < 0.001 | 1.245  (1.110, 1.397) | < 0.001 | 1.312  (1.163, 1.480) | < 0.001 | 1.266  (1.118, 1.434) | < 0.001 |

Model 1, unadjusted model. Model 2, model 1 + adjusted for age, sex. Model 3, model 2 + household income levels, education years, smoking history, medical history (DM, HTN, and DL), BMI, and SBP. Model 4, model 3 + adjusted for fasting glucose, eGFR and dipstick urine protein positivity. CI, confidence interval; OR, odds ratio; Na^+^/Cr, sodium-to-creatinine ratio; Q1, 1^st^ quartile; Q2, 2^nd^ quartile; Q3, 3^rd^ quartile; Q4, 4^th^ quartile.

**Table S4. Binary logistic regression of spot urine Na^+^/Cr levels for the risk of prevalent anemia with additional adjustment for daily iron and protein intake (by 24-hour dietary recall survey) and alcohol consumption**

|  | Spot urine Na^+^/Cr (μmol/mg) | Prevalence, n (%) | Model 1 | | Model 2 | | Model 3 | | Model 4 | |
| --- | --- | --- | --- | --- | --- | --- | --- | --- | --- | --- |
|  |  |  | OR (95%CI) | *P* value | OR (95%CI) | *P* value | OR (95%CI) | *P* value | OR (95%CI) | *P* value |
| Q1 | 2.1 – 54.1 | 925 (5.5) | Reference |  | Reference |  | Reference |  | Reference |  |
| Q2 | 54.1 – 87.2 | 1,126 (7.1) | 1. 311  (1.178, 1.459) | <0.001 | 1.092  (0.978, 1.218) | 0.118 | 1.182  (1.041, 1.342) | 0.011 | 1.228  (1.079, 1.397) | 0.002 |
| Q3 | 87.2 – 136.7 | 1,393 (8.8) | 1.631  (1.479, 1.798) | < 0.001 | 1.121  (1.012, 1.242) | 0.028 | 1.268  (1.121, 1.435) | < 0.001 | 1.345  (1.183, 1.528) | < 0.001 |
| Q4 | 136.7 – 1,344.1 | 1,893 (12.9) | 2.415  (2.192, 2.660) | < 0.001 | 1.225  (1.100, 1.365) | 0.002 | 1.356  (1.189, 1.545) | < 0.001 | 1.469  (1.282, 1.683) | < 0.001 |

Note: Model 1, unadjusted model. Model 2, model 1 + adjusted for age, sex. Model 3, model 2 + household income levels, education years, daily iron and protein intake (by 24-hour dietary recall survey), smoking history, alcohol consumption, medical history (DM, HTN, and DL), BMI, and SBP. Model 4, model 3 + adjusted for fasting glucose, eGFR and dipstick urine protein positivity. Abbreviations CI, confidence interval; OR, odds ratio; Na^+^/Cr, sodium-to-creatinine ratio; Q1, 1^st^ quartile; Q2, 2^nd^ quartile; Q3, 3^rd^ quartile; Q4, 4^th^ quartile.
